# P-1880. Leveraging Pediatric Infectious Diseases Entrustable Professional Activities (EPAs) to Develop and Implement an Innovative Infection Prevention Curriculum

**DOI:** 10.1093/ofid/ofaf695.2049

**Published:** 2026-01-11

**Authors:** Blanca E Gonzalez, Autumn Hayes, Jessica Alban

**Affiliations:** Cleveland Clinic Children's, Cleveland, OH; The Cleveland Clinic, Cleveland, Ohio; The Cleveland Clinic, Cleveland, Ohio

## Abstract

**Background:**

Entrustable Professional Activities (EPAs), defined by the American Board of Pediatrics (ABP), are essential, observable tasks that a general pediatrician must perform safely and effectively to meet patient needs.

A survey of our adult and pediatric Infectious Disease Fellows revealed that the standalone online module, the primary mode of infection prevention (IP) education, was not meeting their educational needs. While it offered foundational knowledge, it lacked practical problem-solving opportunities and expert interaction. To address this gap, we developed a more robust curriculum centered on the EPAs to ensure a more interactive, application-based learning experience.

**Methods:**

Working with both adult and pediatric IPs and using EPA 5 (Prevention and Containment of Infection for Pediatric Infectious Diseases Fellows), we designed a 14-week Infectious Disease/Infection Prevention rotation. The curriculum aligned with the EPA structure and emphasized practical, evidence-based skills in infection prevention and control. It combined structured lectures with hands-on, experiential learning opportunities to reinforce theory and foster real-world application. Examples of lecture topics & tours included but was not limited to, Dialysis and Infection Prevention, Outbreak Investigations, Defining Healthcare-Acquired Infections, tours of water treatment, pharmacy compounding, and sterile processing. At the end of the rotation, fellows provided feedback through a survey to assess the program’s effectiveness and identify areas for improvement.

**Results:**

This program gave fellows valuable insights into the operational aspects of infection prevention. Post-curriculum survey yielded positive feedback for the inclusion of this program within their studies, gratitude for the framework established, and recommendation for modifications to the program which expanded our curriculum from 14 to 16 weeks.

**Conclusion:**

Training in Infection Prevention is crucial for Infectious Disease specialists as it enhances patient safety and quality of care. By aligning the curriculum with ABP’s EPAs, we ensured that fellows developed the competencies needed to address infection control challenges while gaining a deeper understanding of its broader impact on healthcare systems.

**Disclosures:**

All Authors: No reported disclosures

